# Gestational diabetes and adverse perinatal outcomes from 716,152 births in France in 2012

**DOI:** 10.1007/s00125-017-4206-6

**Published:** 2017-02-15

**Authors:** Cécile Billionnet, Delphine Mitanchez, Alain Weill, Jacky Nizard, François Alla, Agnès Hartemann, Sophie Jacqueminet

**Affiliations:** 1 Department of Public Health Studies, Division of Statistics, Strategic Research and Development, National Health Insurance, Paris, France; 2 Division of Neonatology, Department of Perinatology, Armand Trousseau Hospital, APHP, Paris, France; 3 Sorbonne University, UPMC Univ Paris 06, Paris, France; 4 Department of Obstetrics and Gynaecology, Groupe Hospitalier Pitié-Salpêtrière, APHP CNRS UMR 7222, Inserm U1150, Paris, France; 5 Institute of Cardiometabolism and Nutrition, Assistance Publique-Hôpitaux de Paris, Pitié-Salpêtrière Hospital, Paris, France; 6 Diabetes and Metabolic Diseases Department, Pitié-Salpêtrière Hospital, 83 boulevard de l’Hôpital, 75013 Paris, France

**Keywords:** Cardiac malformation, Gestational diabetes mellitus, Macrosomia, Perinatal death, Pre-eclampsia, Preterm

## Abstract

**Aims/hypothesis:**

The aim of this study was to assess the risk of adverse perinatal outcomes in gestational diabetes mellitus (GDM) in a large national cohort.

**Methods:**

All deliveries taking place after 22 weeks in France in 2012 were included by extracting data from the hospital discharge database and the national health insurance system. The diabetic status of mothers was determined by the use of glucose-lowering agents and by hospital diagnosis. Outcomes were analysed according to the type of diabetes and, in the GDM group, whether or not diabetes was insulin-treated.

**Results:**

The cohort of 796,346 deliveries involved 57,629 (7.24%) mothers with GDM. Mother–infant linkage was obtained for 705,198 deliveries. The risks of adverse outcomes were much lower with GDM than with pregestational diabetes. After limiting the analysis to deliveries after 28 weeks to reduce immortal time bias, the risks of preterm birth (OR 1.3 [95% CI 1.3, 1.4]), Caesarean section (OR 1.4 [95% CI 1.4, 1.4]), pre-eclampsia/eclampsia (OR 1.7 [95% CI 1.6, 1.7]), macrosomia (OR 1.8 [95% CI 1.7, 1.8]), respiratory distress (OR 1.1 [95% CI 1.0, 1.3]), birth trauma (OR 1.3 [95% CI 1.1, 1.5]) and cardiac malformations (OR 1.3 [95% CI 1.1, 1.4]) were increased in women with GDM compared with the non-diabetic population. Higher risks were observed in women with insulin-treated GDM than those with diet-treated GDM. After limiting the analysis to term deliveries, an increased risk of perinatal mortality was observed. After excluding women suspected to have undiagnosed pregestational diabetes, the risk remained moderately increased only for those with diet-treated GDM (OR 1.3 [95% CI 1.0, 1.6]).

**Conclusions/interpretation:**

GDM is associated with a moderately increased risk of adverse perinatal outcomes, which is higher in insulin-treated GDM than in non-insulin-treated GDM for most outcomes.

**Electronic supplementary material:**

The online version of this article (doi:10.1007/s00125-017-4206-6) contains peer-reviewed but unedited supplementary material, which is available to authorised users.

## Introduction

The proportions of overweight and obese women are rising in the general population worldwide [1]. Similar trends are observed in France, especially in women of childbearing age [2], thus increasing the risk of type 2 diabetes before pregnancy and the risk of developing gestational diabetes mellitus (GDM) [3]. Moreover, the incidence of diabetes in pregnancy is expected to increase considerably in the future for demographic reasons.

While centre-based studies are likely to reflect the excellence of the reporting centre, the public health perspective requires population-based data to provide solid estimates of complication rates and for making comparisons among countries and populations. Although the consequences of pregestational diabetes have been recognised for a long time, the association between less severe glucose intolerance and morbidity was not definitively proven prior to the publication of findings from the Hyperglycemia and Adverse Pregnancy Outcomes (HAPO) study [4]. However, data on GDM derived from exhaustive national or regional databases that link maternal and neonatal outcomes are relatively rare [5–8], and the overall risk of morbidity of neonates born to GDM mothers remains unclear, particularly for neonates with congenital malformations and respiratory distress [9]. In fact, two problems usually make data interpretation more difficult in GDM studies: (1) the so-called immortal time bias; and (2) the possible contamination of GDM data by inclusion of women with undiagnosed pregestational diabetes, who may be reclassified after pregnancy.

We conducted a large-scale observational national study in France using data for 2012 from the French hospital discharge database and the French National Health Insurance system, which include all deliveries and terminations of pregnancy after 22 weeks for medical reasons. We used a specific algorithm based on the use of glucose-lowering agents before, during and after pregnancy and hospital diagnosis at delivery to determine maternal diabetes status.

The aims of this study were to: (1) estimate the prevalence of GDM in pregnant women in 2012 in France; (2) assess perinatal outcomes after 22 weeks of pregnancy in the presence of GDM compared with pregestational diabetes or no diabetes; (3) determine whether perinatal outcomes differed according to whether GDM was treated by insulin or diet alone; and (4) increase the validity of conclusions by controlling for immortal time bias and contamination by data from women with undiagnosed pregestational diabetes.

## Methods

### Data sources

This cross-sectional study was conducted using combined data from the French hospital discharge database (PMSI [Programme de Médicalisation des Systèmes d’Information]) and the French National Health Insurance database (SNIIRAM [Système National d’Information Inter-Régime de l’Assurance Maladie]). The PMSI database provides detailed medical information on all admissions to French public and private hospitals, including discharge diagnoses using ICD-10 codes (www.who.int/classifications/icd/en/) and medical procedures performed during the hospital stay. The SNIIRAM database contains individualised and anonymous data on health-care claims reimbursed by French National Health Insurance covering the entire French population [10]. Information on severe and costly long-term diseases is also available and coded according to ICD-10. Use of the PMSI and SNIIRAM databases was approved by the French Data Protection Agency (Commission Nationale de l’Informatique et des Libertés).

### Study population

The study included data on all deliveries taking place after 22 weeks of pregnancy in 2012 and recorded in the PMSI database, including terminations of pregnancy for medical reasons. The delivery date was defined as the date of hospital admission for the infant’s birth, when available (88.5%); the date of the delivery procedure, when available (11.4%); or the date of the mother’s hospital admission (0.1%). The date of conception was calculated by using gestational age at delivery as recorded in the database.

A new tool implemented in 2011 links maternal and neonatal data in the PMSI database. In 2012, this tool was available for 88.5% of delivery stays.

### Algorithm for identifying maternal diabetes status

An algorithm for defining the maternal diabetes status was developed for this study based on the use of glucose-lowering agents before, during and after pregnancy, hospital diagnosis at delivery, and information on long-term diseases. The inclusion and exclusion criteria used to identify mothers with type 1 diabetes, type 2 diabetes and GDM are presented in Table 1.

**Table 1:** Criteria used to identify mothers with type 1 and type 2 diabetes and GDM

Criteria	Type 1 diabetes	Type 2 diabetes	GDM
Inclusion	Insulin dispensed at least 3 times in the year before pregnancy AND insulin dispensed at least once from 6 months to 1 year after delivery	Oral glucose-lowering agents or insulin dispensed at least 3 times in the year before pregnancy AND at least one of the following criteria: - an HbA_1c_ assay performed or glucose strips dispensed in the year before pregnancy - long-term disease status for diabetes before pregnancy - oral glucose-lowering agents or insulin dispensed at least once during pregnancy or in the year after delivery	At least one of the following criteria: - insulin dispensed at least once during pregnancy - at least 200 glucose strips dispensed during pregnancy on at least 2 different occasions - a diagnosis of diabetes recorded during the delivery admission (ICD-10 codes E10–E14, O240–O244, O249)^a^
Exclusion	Oral glucose-lowering agents dispensed in the year before pregnancy or the year after delivery	Meeting the definition of type 1 diabetes	Long-term disease status for diabetes before pregnancy Insulin or oral glucose-lowering agents dispensed at least once during the year before pregnancy

^a^ICD-10 codes include both GDM and pregestational diabetes to correct coding errors (GDM that may have been coded as pregestational diabetes), as the sensitivity for GDM observed in the PMSI database was only 73% [16]

GDM was classified as insulin-treated when insulin was dispensed at least once during pregnancy.

As these criteria could not formally exclude mothers with undiagnosed pregestational diabetes from the GDM group, more restrictive criteria were added to exclude those classified as having GDM if insulin or oral glucose-lowering agents had been dispensed at least once during the year after pregnancy.

### Outcomes for mothers

Obstetric outcomes included preterm delivery (<37 weeks of pregnancy), Caesarean section, and discharge diagnosis of eclampsia or pre-eclampsia (ICD-10 codes O140, O141, O149, O15).

### Outcomes for neonates

Neonatal outcomes recorded in this study before the infant’s discharge included macrosomia (birthweight >90th percentile for a given gestational age); Erb’s palsy or clavicle fracture (diagnosis with ICD-10 codes P140, P141, P142, P143 or P134); congenital malformations of the circulatory system (ICD-10 Q20–Q28); congenital malformations of the nervous system (ICD-10 Q00–Q07); perinatal death (including stillbirth and death during the birth stay); birth asphyxia (ICD-10 code P210) and respiratory distress (ICD-10 codes P210, P283, P22 except for P221, P240, P293).

### French recommendations for the screening and treatment of GDM

Since 2010, GDM screening has been recommended when at least one of the following criteria is present: maternal age ≥35 years; BMI ≥25 kg/m^2^; history of diabetes in a first-degree relative; personal history of GDM; and a child with macrosomia [11, 12]. A fasting blood glucose assay (normal <5.1 mmol/l) is recommended during the first trimester. If findings are normal, a 75 g OGTT is indicated at between 24 and 28 weeks of pregnancy. GDM is diagnosed when the fasting blood glucose level is ≥5.1 mmol/l and/or the 1 h blood glucose level is ≥10 mmol/l and/or the 2 h blood glucose level is ≥8.5 mmol/l.

Treatment is based on diet, self-monitoring of blood glucose levels and insulin, when indicated. Other glucose-lowering agents are not used to treat GDM in France.

### Data analysis

Logistic regression models were used to estimate the ORs and 95% CIs for associations between maternal/neonatal outcomes and maternal diabetes status (type 1 diabetes, type 2 diabetes, GDM or no diabetes), adjusted for maternal age (≤29, 30–39, ≥40 years) and birthweight and/or gestational age, depending on the outcomes investigated. Absence of diabetes was used as the reference group to calculate ORs.

A complementary analysis was carried out to study maternal and neonatal outcomes associated with GDM in order to reduce a possible immortal time bias. Immortal time refers to the follow-up time during which, because of the exposure definition, the outcome under study could not occur, corresponding to the time prior to the diagnosis of GDM [13]. As highlighted by Hutcheon et al, differences at the start of follow-up between women with and without GDM can lead to overestimation of the perinatal mortality rate in the population without diabetes [14]. This bias can also affect risk estimation for other outcomes that may occur before the start of the GDM screening period. Consequently, two subgroup analyses were performed to study the risk of all outcomes: one was limited to deliveries after 28 weeks of pregnancy (the recommended screening period for GDM) and the other was limited to deliveries after 37 weeks of pregnancy (at term). Three models were used for each outcome and each subgroup analysis: (1) total GDM compared with no diabetes; (2) insulin-treated and non-insulin-treated GDM compared with no diabetes; and (3) insulin-treated GDM compared with non-insulin-treated GDM. These three models were replicated after excluding undiagnosed type 2 diabetes from GDM.

A *p* value <0.05 was considered to indicate statistical significance. All statistical analyses were performed with SAS software (version 9.2, SAS Institute, Cary, NC, USA).

## Results

Data were available for 796,346 deliveries that occurred in France in 2012. The prevalence of each subtype of diabetes is presented in Table 2. The prevalence of GDM (as defined in Table 1) was 7.24%, ranging from 2.3% in women under the age of 20 years to 16.1% for women over the age of 40 years (electronic supplementary material [ESM] Table 1). Type 2 diabetes accounted for 60% of cases of pre-existing diabetes. Insulin was used to treat 28.1% of women with GDM and 77% of women with type 2 diabetes.

**Table 2:** Distribution of diabetes subtypes among deliveries occurring after 22 weeks in the French population in 2012

Diabetes status	Women, *n* (%)	Maternal age, y (mean ± SD)
Type 1 diabetes	1291 (0.16)	30.0 ± 5.5
Type 2 diabetes	1907 (0.24)	33.5 ± 5.5
GDM	57,629 (7.24)	31.9 ± 5.5
No diabetes	735,519 (92.34)	29.5 ± 5.3
Total	796,346 (100.00)	29.7 ± 5.4

Including terminations of pregnancies performed after 22 weeks

y, years

Linked maternal and neonatal data were available for 705,198 deliveries (88.5%), corresponding to 716,152 neonates. The prevalence of GDM was 6.7% in the group of mothers for whom neonatal data were not available.

Maternal and neonatal outcomes among all deliveries are presented in Table 3. The risks of Caesarean section, eclampsia/pre-eclampsia and preterm birth were higher in the GDM group than in the no diabetes group. The risks of all adverse neonatal outcomes studied were also significantly increased in the presence of GDM, except for the risk of nervous system malformations (which was unchanged) and the risk of death (which was significantly decreased). Compared with the pregestational diabetes groups, the risks of adverse maternal and neonatal outcomes were lower in the GDM group.

**Table 3:** Risk of maternal and perinatal outcomes by maternal diabetes status

Outcome	No diabetes	GDM	T1D	T2D
Maternal (*n* = 796,346)				
*n*	735,519	57,629	1291	1907
Caesarean section				
Rate, %	19.6	27.8	57.1	50.6
OR (95% CI)^a^	1	1.4 (1.4, 1.5)	4.3 (3.8, 4.8)	3.2 (2.9, 3.5)
Eclampsia/pre-eclampsia				
Rate, %	1.6	2.6	9.6	6.4
OR (95% CI)^b^	1	1.6 (1.5, 1.7)	6.6 (5.5, 8.0)	4.0 (3.3, 4.8)
Delivery at <37 weeks				
Rate, %	7.0	8.4	30.4	19.0
OR (95% CI)^b^	1	1.2 (1.2, 1.3)	5.8 (5.2, 6.6)	3.1 (2.7, 3.4)
Neonatal (*n* = 716 152)				
*n*	660,867	52,488	1120	1677
Perinatal death^c^				
Rate, %	0.6	0.5	1.2	2.4
OR (95% CI)^b^	1	0.7 (0.6, 0.8)	1.8 (1.0, 3.1)	3.6 (2.6, 5.0)
Asphyxia				
Rate, %	0.9	1.0	3.3	2.0
OR (95% CI)^b^	1	1.2 (1.1, 1.3)	3.9 (2.8, 1.3)	2.4 (1.7, 3.3)
Macrosomia				
Rate, %	9.2	15.7	43.7	28.9
OR (95% CI)^b^	1	1.8 (1.7, 1.8)	7.7 (6.8, 8.6)	3.8 (3.4, 4.2)
Erb’s palsy/clavicle fracture^d^				
Rate, %	0.5	0.7	2.0	1.5
OR (95% CI)^e^	1	1.3 (1.1, 1.5)	3.7 (1.9, 6.9)	2.7 (1.6, 4.7)
Cardiac malformations				
Rate, %	0.7	0.9	3.8	2.8
OR (95% CI)^b^	1	1.2 (1.1, 1.3)	5.3 (3.9, 7.2)	3.8 (2.8, 5.1)
Nervous system malformations				
Rate, %	0.15	0.12	0.36	0.42
OR (95% CI)^b^	1	0.8 (0.6, 1.0)	2.3 (0.9, 6.2)	2.7 (1.3, 5.8)
Respiratory distress				
Rate, %	2.9	3.6	11.4	7.3
OR (95% CI)^a^	1	1.3 (1.2, 1.3)	2.1 (1.7, 2.6)	1.7 (1.4, 2.1)

^a^Adjusted for maternal age and gestational age

^b^Adjusted for maternal age

^c^Calculated on the 713,750 deliveries excluding terminations of pregnancy after 22 weeks

^d^Calculated on the 570,171 deliveries excluding Caesarean sections

^e^Adjusted for maternal age and birthweight

T1D, type 1 diabetes; T2D, type 2 diabetes

In order to avoid immortal time bias, the analysis was limited to deliveries after 28 weeks. We also conducted an analysis of deliveries after 37 weeks to determine the risk of adverse outcomes in term deliveries. In each subgroup, maternal and neonatal outcomes were compared with the population without diabetes, and according to whether or not diabetes was insulin-treated (Tables 4 and 5).

**Table 4:** Risk for maternal and neonatal outcomes among deliveries occurring after 28 weeks in the GDM group by diabetes treatment

Outcome	No diabetes	GDM	Insulin-treated GDM	Non-insulin-treated GDM
Deliveries >28 weeks				
*n*	729,105	57,383	16,108	41,275
Caesarean section				
Rate, %	19.5	27.8	34.0	25.3
OR (95% CI)^a^	1	1.4 (1.4, 1.4)	1.7 (1.7, 1.8)	1.3 (1.2, 1.3)
Eclampsia/pre-eclampsia				
Rate, %	1.5	2.5	2.4	2.6
OR (95% CI)^b^	1	1.7 (1.6, 1.7)	1.6 (1.4, 1.7)	1.7 (1.6, 1.8)
Delivery <37 weeks				
Rate, %	6.1	8.0	9.2	7.6
OR (95% CI)^b^	1	1.3 (1.3, 1.4)	1.5 (1.4, 1.6)	1.2 (1.2, 1.3)
Neonatal				
*n*	655,534	52,279	14,781	37,498
Perinatal death^c^				
Rate, %	0.32	0.36	0.35	0.36
OR (95% CI)^b^	1	1.1 (0.9, 1.3)	1.0 (0.8, 1.4)	1.1 (0.9, 1.3)
Asphyxia				
Rate, %	0.8	1.0	0.9	1.0
OR (95% CI)^b^	1	1.2 (1.1, 1.3)	1.1 (0.9, 1.3)	1.2 (1.1, 1.4)
Macrosomia				
Rate, %	9.2	15.7	18.5	14.5
OR (95% CI)^b^	1	1.8 (1.7, 1.8)	2.1 (2.1, 2.2)	1.6 (1.6, 1.7)
Erb’s palsy/clavicle fracture^d^				
Rate, %	0.5	0.7	0.7	0.7
OR (95% CI)^e^	1	1.3 (1.1, 1.5)	1.4 (1.1, 1.8)	1.2 (1.1, 1.4)
Cardiac malformations				
Rate, %	0.6	0.8	1.1	0.7
OR (95% CI)^b^	1	1.3 (1.1, 1.4)	1.7 (1.4, 2.0)	1.1 (1.0, 1.3)
Nervous system malformations				
Rate, %	0.12	0.11	0.08	0.12
OR (95% CI)^b^	1	0.9 (0.7, 1.2)	0.7 (0.4, 1.2)	1.0 (0.8, 1.4)
Respiratory distress				
Rate, %	2.7	3.4	3.5	3.4
OR (95% CI)^a^	1	1.1 (1.0, 1.3)	1.4 (1.2, 1.7)	1.0 (0.9, 1.2)

^a^Adjusted for maternal age and gestational age

^b^Adjusted for maternal age

^c^Calculated on deliveries excluding terminations of pregnancy after 22 weeks

^d^Calculated on deliveries excluding Caesarean sections

^e^Adjusted for maternal age and birthweight

**Table 5:** Risk for maternal and neonatal outcomes among deliveries occurring after 37 weeks in the GDM group by diabetes treatment

Outcome	No diabetes	GDM	Insulin-treated GDM	Non-insulin-treated GDM
Maternal				
*n*	684,398	52,780	14,633	38,147
Caesarean section				
Rate, %	18.3	26.2	32.7	23.8
OR (95% CI)^a^	1	1.4 (1.4, 1.4)	1.8 (1.7, 1.9)	1.3 (1.2, 1.3)
Eclampsia/pre-eclampsia				
Rate, %	1.0	1.7	1.6	1.7
OR (95% CI)^b^	1	1.7 (1.6, 1.8)	1.6 (1.4, 1.8)	1.7 (1.6, 1.9)
Neonatal				
*n*	614,853	47,959	13,403	34,556
Perinatal death^c^				
Rate, %	0.15	0.21	0.21	0.21
OR (95% CI)^b^	1	1.3 (1.1, 1.6)	1.3 (0.9, 1.9)	1.3 (1.1, 1.7)
Asphyxia				
Rate, %	0.7	0.8	0.8	0.9
OR (95% CI)^b^	1	1.2 (1.1, 1.3)	1.1 (0.9, 1.3)	1.2 (1.1, 1.4)
Macrosomia				
Rate, %	9.2	15.6	18.2	14.6
OR (95% CI)^b^	1	1.8 (1.7, 1.8)	2.1 (2.0, 2.2)	1.6 (1.6, 1.7)
Erb’s palsy/clavicle fracture^d^				
Rate, %	0.5	0.7	0.7	0.7
OR (95% CI)^e^	1	1.3 (1.1, 1.5)	1.4 (1.1, 1.8)	1.2 (1.1, 1.4)
Cardiac malformations				
Rate, %	0.50	0.68	0.96	0.57
OR (95% CI)^b^	1	1.3 (1.2, 1.5)	1.9 (1.5, 2.2)	1.1 (1.1, 1.3)
Nervous system malformations				
Rate, %	0.08	0.08	0.07	0.08
OR (95% CI)^b^	1	1.0 (0.7, 1.4)	1.0 (0.5, 1.8)	1.0 (0.7, 1.5)
Respiratory distress				
Rate, %	1.6	2.0	2.2	2.0
OR (95% CI)^a^	1	1.3 (1.1, 1.4)	1.7 (1.4, 2.1)	1.1 (0.9, 1.3)

^a^Adjusted for maternal age and gestational age

^b^Adjusted for maternal age

^c^Calculated on deliveries excluding terminations of pregnancy after 22 weeks

^d^Calculated on deliveries excluding Caesarean sections

^e^Adjusted for maternal age and birthweight

For deliveries after 28 weeks, risks for maternal and neonatal outcomes in the presence of GDM were similar to those reported in Table 3, except that the risk of respiratory distress was lower (OR 1.1 [95% CI 1.0, 1.3] vs OR 1.3 [95% CI 1.2, 1.3]) and the risk of perinatal death, which was no longer decreased compared with the no diabetes group.

The risks of Caesarean section, delivery before 37 weeks and macrosomia were higher in the insulin-treated GDM group than in the non-insulin-treated GDM group. The excess risk of cardiac malformations and respiratory distress observed in the GDM group was also due to the insulin-treated GDM group.

In deliveries after 37 weeks of pregnancy, we found a 30% increase in the OR for perinatal death in the GDM group compared with the no diabetes group. This risk was similar whether or not the diabetes was insulin-treated. No significant differences were observed for the other outcomes in deliveries after 28 weeks.

As the unexpected increased risk of mortality at term observed in the GDM group might be due to undiagnosed pregestational diabetes in this group, we repeated the analyses using more restrictive criteria to exclude women classified as having GDM but to whom insulin or oral glucose-lowering agents were dispensed at least once during the year after pregnancy. This analysis excluded 1376 women in the group of deliveries after 28 weeks (6.8% in the insulin-treated group and 0.7% in the non-insulin-treated group), and 1171 women in the group of deliveries after 37 weeks (7.3% in the insulin-treated group and 0.64% in the non-insulin-treated group). The characteristics of these women are presented in ESM Table 2.

In this restricted GDM group, the risk of respiratory distress among deliveries after 28 weeks (ESM Table 3) and the risk of perinatal death among deliveries after 37 weeks (ESM Table 4) in the insulin-treated group were no longer significantly increased (OR 1.0 [95% CI 0.9, 1.1] and OR 0.9 [95% CI 0.6, 1.5], respectively). However, the risk of perinatal death among deliveries after 37 weeks remained moderately increased in the non-insulin-treated group (OR 1.3 [95% CI 1.0, 1.6]). Table 6 summarises the outcomes that were significantly increased in the insulin-treated group compared with the non-insulin-treated group.

**Table 6:** Outcomes that were significantly increased in the insulin-treated GDM group

Outcome^a^	OR (95% CI)^b^
Delivery at >28 weeks	
Delivery at <37 weeks	1.2 (1.1, 1.2)
Caesarean section	1.4 (1.3, 1.4)
Macrosomia	1.3 (1.2, 1.4)
Cardiac malformation	1.4 (1.1, 1.7)
Delivery at ≥37 weeks	
Caesarean section	1.4 (1.3, 1.4)
Macrosomia	1.3 (1.2, 1.3)
Cardiac malformation	1.6 (1.2, 2.0)

Data excluded mothers to whom insulin or oral glucose-lowering agents were dispensed during the year after pregnancy

^a^Only outcomes with significant ORs (*p <* 0.05) are included

^b^Compared with the non-insulin-treated GDM group

**Fig. 1:**
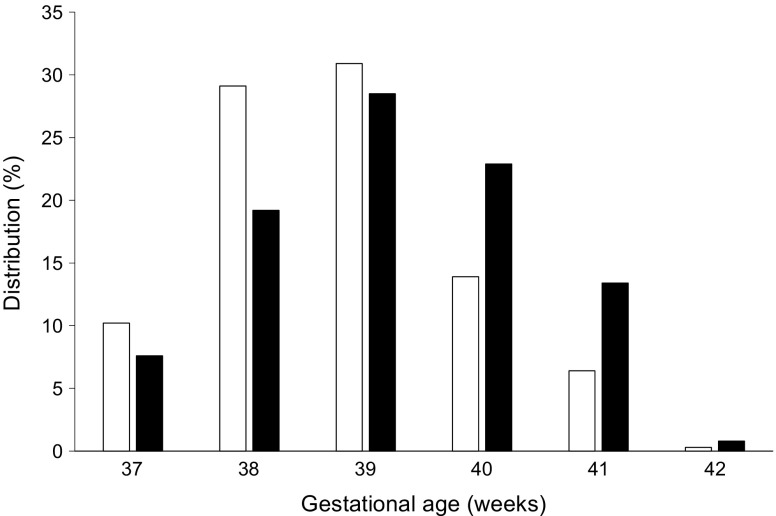
Distribution of gestational age at delivery in the GDM group, according to maternal treatment and restricted to deliveries that occurred after 37 weeks of pregnancy. White bars, insulin-treated GDM; black bars, non-insulin-treated GDM

Finally, we hypothesised that the risk of death in the non-insulin-treated group may be related to a later term of delivery compared with the insulin-treated group. We therefore compared the distribution of gestational age at delivery in the group of deliveries after 37 weeks according to maternal treatment. Women with GDM who did not receive insulin treatment delivered later than those who did (Fig. 1). Similar values were observed after excluding women to whom insulin or glucose-lowering agents were dispensed during the year following delivery.

## Discussion

On the basis of the analysis of data for 2012 obtained from a large-scale, nationwide, exhaustive database in France, we confirm that GDM is associated with lower risks for maternal and neonatal complications compared with pregestational diabetes. However, we show that: (1) the risk of cardiac malformations is increased for women with insulin-treated GDM, whereas the risk of nervous system malformations is not; (2) the risk of respiratory distress is also increased for this subgroup of insulin-treated women with GDM; and (3) the risk of perinatal mortality increases with GDM for deliveries after 37 weeks.

This is the first nationwide study to evaluate the effect of diabetes on pregnancy in France based on a combination of maternal and neonatal data. The strengths of our study are that we had access to data concerning 796,346 deliveries and combined maternal and neonatal data for 88.5% of these deliveries, and that women were accurately classified with various types of diabetes during pregnancy. This classification allowed us to analyse outcomes in a large GDM cohort according to whether or not diabetes was insulin-treated and in a second analysis to avoid ‘contamination’ of GDM data by the presence of women with undiagnosed pregestational diabetes. Furthermore, by taking the immortality bias into account (which is not usually done), we considerably increased the robustness of our results. We also analysed the outcomes in term pregnancies (≥37 weeks) in order to alert clinic and primary hospital physicians to risks potentially related to GDM at term.

A weakness of our study is that we did not have access to data for 11.5% of neonates. Another limitation is the absence of data on glycaemic control in patients and other comorbidities such as maternal BMI.

The validity of the PMSI database has been regularly audited, especially in terms of perinatal data [15, 16]: prematurity and Caesarean section, for example, are well documented. However, data on maternal morbidity appear to be less comprehensive, as indicated by a sensitivity of 73% for GDM. Coding errors concerning the diagnosis of diabetes in the PMSI database were therefore corrected by the algorithm based on medication use.

The prevalence of GDM in France in 2012 was 7.24%, which was lower than that reported in other countries: recently reported GDM prevalence rates of 15% in the USA [17] and 13% in Australia [18] were similar to those observed in the HAPO study population [19]. However, in France, experts recommend restricting GDM screening to women presenting with certain risk factors, which could explain the lower prevalence in this study [11].

In a recent large-scale, nationwide Danish cohort, the risk of cardiac malformations was moderately increased in GDM pregnancies [20]. We found similar results, but showed that this increase was significant only in women with insulin-treated GDM. The excess risk of cardiac malformations observed in this group persisted after the exclusion of women with undiagnosed pregestational diabetes, indicating that mechanisms other than periconceptional maternal blood glucose levels may increase the risk of cardiac malformations [21]. In addition to the severity of diabetes, pre-pregnancy BMI in mothers with GDM was previously shown to be a predictor of congenital malformations in infants [22]. Other teams have shown that high maternal BMI is associated with an increased risk of malformations that is independent of maternal blood glucose levels [23, 24]. We can hypothesise that the increased risk of cardiac malformations in women with insulin-treated GDM may be partly associated with maternal obesity, although maternal BMI was not recorded in the national database used for this study. We found that GDM did not alter the risk of nervous system malformations. The incidence of these malformations was much lower than the incidence of cardiac malformations, as previously reported by other authors [22, 25]. One hypothesis is that early hyperglycaemic exposure of the embryo has different teratogenic effects on cardiac and nervous system tissues. Nervous system malformations may also be associated with an increased risk of miscarriage or a higher rate of termination of pregnancy before 22 weeks, which was not taken into account in this study.

The relationship between maternal GDM and the risk of neonatal respiratory distress has not been clearly established [26]. Data from our study clearly suggest an increased risk of neonatal respiratory distress in the insulin-treated GDM group, and this risk was also increased for deliveries after 37 weeks. It is likely that, in the setting of very preterm delivery, lung immaturity remains the leading risk factor for respiratory distress irrespective of the presence or absence of diabetes in pregnancy. On the other hand, poorly managed maternal diabetes has been shown to be associated with delayed appearance of phosphatidylglycerol (a main compound of pulmonary surfactant) in amniotic fluid after 34 weeks of pregnancy [27]. Target glycaemic levels are often more difficult to attain in insulin-treated women with GDM than in diet-treated women. A recent study reported that insulin-treated diabetes was an independent risk factor for respiratory distress in neonates born after 33 weeks in a group of women with pregestational diabetes or GDM [28].

Whether or not GDM is associated with an increased risk of perinatal mortality remains a controversial subject. Most recent studies have indicated the absence of increased risk [5, 8], but these studies did not take immortality bias into account. In the study by Hutcheon et al [14], the RR for stillbirth among women with GDM was 1.25 in analyses limited to births after 28 weeks. In our study, the risk of perinatal mortality was not increased among deliveries after 28 weeks: surprisingly, it was increased only among deliveries after 37 weeks, whether or not women were insulin-treated. However, the 30% increase in RR remains proportionally low: 0.21% in GDM deliveries vs 0.15% in no diabetes deliveries. After excluding women with undiagnosed pregestational diabetes, the risk of perinatal death in deliveries after 37 weeks in the insulin-treated group was no longer increased, although excess risk persisted in the non-insulin-treated group. Cundy et al reported that, after excluding newly diagnosed type 2 diabetes from the GDM group, the perinatal mortality rate was no longer increased [29]. The increased risk of perinatal mortality observed in the group of women with GDM treated exclusively by diet is more difficult to explain. Perinatal deaths could be secondary to longer exposure to hyperglycaemia, as we found that non-insulin-treated women with GDM had a later term of delivery compared with insulin-treated women. Other authors have shown that women with GDM are more likely than women without diabetes to experience stillbirth after 35 weeks, suggesting the existence of a mortality benefit in delivering women with GDM at 39 weeks instead of continuing with expectant management [30].

### Conclusion

We have clearly demonstrated that GDM is a disease related to adverse pregnancy outcomes and that most of the risks are higher in women with insulin-treated GDM. By restricting analysis to deliveries after 37 weeks and excluding cases of undiagnosed pregestational diabetes, we identified a moderate increase in perinatal mortality in non-insulin-treated women with GDM. Although more investigation is needed, this study helps illuminate the controversy about timing of delivery in GDM pregnancy.

## Electronic supplementary material

Below is the link to the electronic supplementary material.

ESM 1 (PDF 21.5 kb)

## Abbreviations

GDM: Gestational diabetes mellitus
HAPO: Hyperglycemia and Adverse Pregnancy Outcomes
PMSI: French hospital discharge database (Programme de Médicalisation des Systèmes d’Information)
SNIIRAM: French National Health Insurance database (Système National d’Information Inter-Régime de l’Assurance Maladie)
