# Supplementary material for: Gestational diabetes and adverse perinatal outcomes from 716,152 births in France in 2012
Source: Diabetologia. 2017 Feb 15;60(4):636–44. doi: 10.1007/s00125-017-4206-6 (PMC6518373; doi:10.1007/s00125-017-4206-6)
Supplement: Supplementary file 1 — (PDF 21.5 kb) [file 125_2017_4206_MOESM1_ESM.pdf]

Table 1 ESM: Number and percentage of women who delivered after 22 weeks (including terminations of pregnancy after 22 weeks) for each age-group by diabetes status

Maternal age (years)	No diabetes	GDM	T1D	T2D	Total
≤ 19					
n	19,480	450	30	10	19,970
Rate, %	97.5	2.3	0.2	0.1	
20-24					
n	110,487	4746	173	100	115,506
Rate, %	95.7	4.1	0.1	0.1	
25-29					
n	239,608	14,445	407	330	254,790
Rate, %	94.0	5.7	0.2	0.1	
30-34					
n	233,762	19,199	422	641	254,024
Rate, %	92.0	7.6	0.2	0.3	
35-39					
n	106,912	13,865	193	546	121,516
Rate, %	88.0	11.4	0.2	0.4	
≥ 40					
n	25,270	4924	66	280	30,540
Rate, %	82.7	16.1	0.2	0.9	
Total	735,519	57,629	1291	1907	796,346

T1D, type 1 diabetes; T2D, type 2 diabetes

Table 2 ESM: Maternal and neonatal outcomes among deliveries of mothers excluded from the GDM group according to the restrictive criteria (see material and method section).

	Deliveries >28 weeks	Deliveries ≥37 weeks
Maternal		
n	1376	1171
Caesarean section		
Rate, %	38.88	36.38
Eclampsia/Preeclampsia		
Rate, %	4.58	2.65
Delivery <37 weeks		
Rate, %	14.90	NA
Treated by insulin during pregnancy		
Rate, %	78.78	79.16
Neonatal		
n	1221	1036
Perinatal death[a]		
Rate, %	2.21	1.25
Asphyxia		
Rate, %	1.56	1.54
Macrosomia		
Rate, %	27.52	27.12
Erb's palsy/clavicle fracture[b]		
Rate, %	0.92	1.05
Cardiac malformations		
Rate, %	2.29	1.74
Nervous system malformations		
Rate, %	0.25	0.19
Respiratory distress		
Rate, %	6.39	4.05

[a] Calculated on deliveries excluding terminations of pregnancy after 22 weeks
[b] Calculated on deliveries excluding caesarean sections
NA: not applicable

Table 3 ESM: Risk for maternal and neonatal outcomes among deliveries after 28 weeks in the GDM group by diabetes treatment

Outcome		No diabetes	GDM	Insulin-treated GDM	Non-insulin-treated GDM
Maternal					
n		729,105	56,007	15,024	40,983
Caesarean section					
	Rate, %	19.5	27.5	33.5	25.3
	OR (95% CI) [a]	1	1.4 (1.3, 1.4)	1.7 (1.6, 1.8)	1.3 (1.2, 1.3)
Eclampsia/preeclampsia					
	Rate, %	1.5	2.5	2.3	2.6
	OR (95% CI) [b]	1	1.6 (1.5, 1.7)	1.5 (1.3, 1.6)	1.7 (1.6, 1.8)
Delivery <37 weeks					
	Rate, %	6.1	7.9	8.8	7.5
	OR (95% CI) [b]	1	1.3 (1.2, 1.3)	1.4 (1.4, 1.5)	1.2 (1.2, 1.3)
Neonatal					
n		655,534	51,058	13,825	37,233
Perinatal death[c]					
	Rate, %	0.32	0.31	0.24	0.34
	OR (95% CI) [b]	1	0.9 (0.8, 1.1)	0.7 (0.5, 1.0)	1.0 (0.9, 1.2)
Asphyxia					
	Rate, %	0.8	1.0	0.9	1.0
	OR (95% CI) [b]	1	1.2 (1.1, 1.3)	1.1 (0.9, 1.3)	1.2 (1.1, 1.4)
Macrosomia					
	Rate, %	9.2	15.4	18.0	14.4
	OR (95% CI) [b]	1	1.7 (1.7, 1.8)	2.1 (2.0, 2.2)	1.6 (1.6, 1.7)
Erb's palsy/clavicle fracture[d]					
	Rate, %	0.5	0.7	0.7	0.7
	OR (95% CI) [e]	1	1.3 (1.1, 1.5)	1.4 (1.1, 1.8)	1.2 (1.1, 1.4)
Cardiac malformations					
	Rate, %	0.62	0.76	0.96	0.69
	OR (95% CI) [b]	1	1.2 (1.1, 1.3)	1.5 (1.3, 1.8)	1.1 (1.0, 1.2)
Nervous system malformations					
	Rate, %	0.12	0.11	0.08	0.12
	OR (95% CI) [b]	1	0.9 (0.7, 1.2)	0.7 (0.4, 1.2)	1.0 (0.6, 1.4)
Respiratory distress					
	Rate, %	2.7	3.3	3.3	3.4
	OR (95% CI) [a]	1	1.1 (1.05 , 1.2)	1.0 (0.9, 1.1)	1.1 (1.1, 1.2)

Data excluded mothers to whom insulin or oral glucose-lowering agents were prescribed during the year after pregnancy.

[a] Adjusted for maternal age and gestational age

[b] Adjusted for maternal age

[c] Calculated on deliveries excluding terminations of pregnancy after 22 weeks

[d] Calculated on deliveries excluding caesarean sections

[e] Adjusted for maternal age and birthweight

Table 4 ESM: Risk for maternal and neonatal outcomes among deliveries after 37 weeks in the GDM group by diabetes treatment.

Outcome		No diabetes	GDM	Insulin-treated GDM	Non-insulin-treated GDM
Maternal					
n		684,398	51,609	13,706	37,903
Caesarean section					
	Rate, %	18.3	26.0	32.2	23.8
	OR (95% CI) [a]	1	1.4 (1.4, 1.4)	1.8 (1.7, 1.8)	1.3 (1.2, 1.3)
Eclampsia/ preeclampsia					
	Rate, %	1.0	1.6	1.5	1.7
	OR (95% CI) [b]	1	1.7 (1.6, 1.8)	1.5 (1.3, 1.8)	1.7 (1.6, 1.9)
Neonatal					
n		614,853	46,923	12,586	34,337
Perinatal death[c]					
	Rate, %	0.15	0.19	0.15	0.20
	OR (95% CI) [b]	1	1.2(0.9, 1.5)	0.9 (0.6, 1.5)	1.3 (1.0, 1.6)
Asphyxia					
	Rate, %	0.7	0.8	0.8	0.8
	OR (95% CI) [b]	1	1.2 (1.05, 1.3)	1.1 (0.9, 1.3)	1.2 (1.1, 1.3)
Macrosomia					
	Rate, %	9.2	15.3	17.6	14.5
	OR (95% CI) [b]	1	1.7 (1.7, 1.8)	2.0 (1.9, 2.1)	1.6 (1.6, 1.7)
Erb's palsy/clavicle fracture[d]					
	Rate, %	0.5	0.7	0.7	0.7
	OR (95% CI) [e]	1	1.3 (1.1, 1.4)	1.4 (1.1, 1.8)	1.2 (1.1, 1.4)
Cardiac malformations					
	Rate, %	0.50	0.65	0.90	0.56
	OR (95% CI) [b]	1	1.3 (1.1, 1.4)	1.7 (1.4, 2.1)	1.1 (1.0, 1.3)
Nervous system malformations					
	Rate, %	0.08	0.08	0.07	0.08
	OR (95% CI) [b]	1	1.0 (0.7, 1.4)	1.0 (0.5, 1.8)	1.0 (0.7, 1.5)
Respiratory distress					
	Rate, %	1.6	2.0	2.1	2.0
	OR (95% CI) [a]	1	1.2 (1.2, 1.3)	1.3 (1.1, 1.5)	1.2 (1.1, 1.3)

Data excluded mothers to whom insulin or oral glucose-lowering agents were prescribed during the year after pregnancy.

[a] Adjusted for maternal age and gestational age

[b] Adjusted for maternal age

[c] Calculated on deliveries excluding terminations of pregnancy after 22 weeks

[d] Calculated on deliveries excluding caesarean sections

[e] Adjusted for maternal age and birthweight
